# Commentary on the Organisation of Occupational Health and Safety in Southern Africa, the International Labour Organization and Policies in General

**DOI:** 10.29024/aogh.2333

**Published:** 2018-08-31

**Authors:** France Ncube, Artwell Kanda

**Affiliations:** 1 Department of Environmental Science, Bindura University of Science Education, Private Bag 1020, Bindura, ZW

## Abstract

**Background:**

The design and implementation of sound occupational health and safety (OHS) programmes require understanding of the main issues that need attention. This article highlights key issues regarding the (i) organisation of OHS services in southern Africa, (ii) role of the International Labour Organization (ILO) in the provision of OHS services and (iii) implementation of policies in general.

**Methods:**

Relevant peer-reviewed journal articles, ILO conventions and policies were identified and discussed.

**Results:**

Inadequacies that exist on the organisation of OHS services in southern Africa include (i) lack of some critical categories of OHS practitioners, (ii) no emphasis on the surveillance of the work environment (iii) disregard of the worker’s right to refuse to work in unsafe work environments and (iv)non-coverage of some sectors of the economy. Further research is needed to identify additional efforts that the ILO requires to effectively discharge its OHS promotion mandate.

**Conclusion:**

Responsible authorities need to attend to the shortcomings of the national OSH laws and intergovernmental pacts.

On February 17, 1988, the International Labour Organization’s (ILO) Convention 161 [1] came into effect. This convention focuses on the organisation of occupational health services and addresses issues such as the need for the formulation, implementation and review of occupational health and safety (OHS) policies in member states. The present article comments on the organisation of OHS in southern Africa [2], the International Labour Organization (ILO) and policies in general.

## Occupational Health and Safety Organization in Southern Africa [2]

In the four regions of Africa, very few research efforts have been focused on examining existing OHS legislation in order to suggest required improvements for actioning by policy makers. However, in the *Annals of Global Health*, Moyo and colleagues [2] make a valuable contribution in this regard, in the context of southern Africa. More importantly, the publication of their findings in an open access scientific journal is a giant step towards drawing the attention of OHS practitioners, policy makers and the scientific community to the fundamental concerns requiring correction. Given the value of the conclusions arising from their work, Moyo and colleagues [2] may also need to consider additional platforms for sharing their findings, such as the annual Public Health Practitioners Association Conference (PHASA), hosted by South Africa, which is regularly well attended by researchers, policy makers and OHS practitioners from various developing and developed countries. On the other hand, the authors’ country selection may not necessarily mirror the status of OHS laws in the remaining unreviewed majority of other southern African countries. Consequently, further work may need to also concentrate on such nations as well as the eastern, western and northern African countries, in order to identify their peculiar scenarios requiring redress.

About two decades back, Auria [3] rightly observed that the bulk of published OHS articles were unfortunately small-scale, local and not national and strategic. Fundamentally, Moyo and colleagues’ regional study is a step ahead rather than repetition of previous research shortcomings raised by Auria [3]. In their study, Moyo et al. [2] describe, discuss and analyse OHS laws in South Africa, Zimbabwe, Botswana and Zambia and observe, among other issues, the severe shortage of occupational medicine specialists. Similar human resource challenges stifling the performance of OHS services have been reported in various other developing countries [4,5,6]. Although massive improvements in such critical expert staffing levels in resource-constrained countries might appear difficult, some positive improvements in African countries, such as South Africa, fruitfully demonstrate that these limitations are solvable. Consequently, the need for researchers’ sustained efforts in convincing policy makers to take practical remedial action is more crucial than ever. Further delays in the rectification of the OHS expertise deficits prevailing in various developing countries are unwelcome giant steps backwards that are grossly incongruent with the requirements in article 5 of the ILO convention 161 of 1985. Article 5 requires organisations to provide vital OHS functions such as risk assessment, workplace and worker’s health surveillance, expert guidance, ergonomics and first aid and emergency treatment.

Moyo and colleagues rightly bemoan the lack of coverage of informal workers, the civil service and domestic workers in reviewed compensation laws. The economies of developing countries appear to be driven by the informal sector, which suggests that informal workers, like their formally employed counterparts in the manufacturing and mining industries, deserve top priority with regard to OHS services provision. Similarly, in industrialised countries, the work conditions of informal workers seem not to meet the fundamentals of decent work. For instance, some studies report that informal workers: (1) do not have social security coverage [7], (2) require consideration with regard to enforcement of universal labour rights [8] and (3) are vulnerable to various work-related health problems [7,8,9]. Therefore, in both developing and industrialised countries, the lack of coverage of the informal sector by existing OHS laws is a disheartening omission that suggests that their workplace injuries and unsafe conditions largely go unmonitored, unreported and uninvestigated. We call upon policy makers to broaden coverage of OHS services to such currently neglected sectors.

A central theme emerging from the Moyo et al. [2] study is the finding that OHS functions are fragmented among the ministries of health, labour and mining. The authors provide a detailed and comprehensive evidence-based analysis of particular OHS laws administered by different government departments in reviewed countries. However, some critical issues remain unresolved and unaddressed and deserve further examination, such as how such fragmentation can impinge on enforcement and how it can possibly be corrected. From an economic perspective, fragmentation may result in duplication of enforcement roles of OHS laws, especially where there are overlaps, which may result in costly waste of scarce resources. Still, fragmentation may yield inconsistencies and lack of uniformity on the implementation of enforcement functions among concerned departments. These limitations may hamper progress concerning safeguarding workers’ health. Therefore, more efforts are required with regard to exploring options for solving the fragmentation, such as unifying the laws under one regulatory authority.

Moyo and colleagues wonderfully present the strengths of Zimbabwe’s Pneumoconiosis Act [10]. The provision for various medical examinations of workers exposed to dusty occupations is stressed. As such, the examinations constitute a necessary proactive surveillance approach for worker fitness screening and for early identification of signs of ill health in those workers in dusty occupations. However, two fundamental points are missing in the article and deserve the urgent attention of policy makers. Firstly, the Act only certifies the worker fit to work in a dusty environment but neither certifies the work environment fit for the worker nor obliges the employer to improve conditions of work in dusty occupations. Secondly, an overwhelming shortcoming of the Act is that it does not give workers the right to refuse to work under unsafe and unhealthy work environments. Therefore, the Act appears predominantly employer-centric rather than worker-centric. Taken together, these omissions of the Act are an unfortunate misstep grossly inconsistent with ILO Conventions ratified by Zimbabwe. For example, Convention 161 requires employers to offer workplace risk assessment whilst the Convention 170 on chemical safety [11] contains the worker’s right to refuse to work in unsafe environments.

## ILO and Policies in General

Most sub-Saharan countries are predominantly agrarian [12] but lack laws for protecting the OHS of agricultural workers. Agricultural work may expose workers to diverse synthetic pesticides and herbicides which may be genotoxic, mutagenic, carcinogenic, neurotoxic and endocrine-disrupting. At the global level, the ILO 170 governs the safe manufacture, labelling, transportation, use, handling and disposal of chemicals. Importantly, this convention appears insufficient with regards to sound protection of agricultural workers’ occupational health. It does not, for example, ban usage of the synthetic pesticides and herbicides in the sector, despite the emergence of overwhelming evidence on the availability of equally effective alternative non-chemical agricultural pest control methods like organic farming and integrated pest control initiatives.

Moreover, ILO seems grossly limited to the production of OHS proposals for implementation by member states, particularly, conventions and recommendations. Since ILO lacks the mandate for direct OHS lawmaking, its conventions merely serve as draft legislation ultimately requiring both ratification and subsequent enactment into locally binding national OHS law by member states. Worse still, recommendations are not designed to be legally binding but to instead provide optional guidelines concerning OHS policies, laws and practice. Therefore, beyond conventions and recommendations, further research may need to identify additional efforts that ILO requires to effectively discharge its OHS promotion mandate.

On the other hand, the ILO conventions anchoring decent work concerns have several missing components, which make them less relevant in the OHS field. For example, the ILO’s Convention for Minimum Age on Child Labour lacks the much needed specific standard definition of what constitute a minimum working age. Without such a definition, member states are bound to adopt different minimum ages for working, which will make it a complicated and daunting task for ILO to effectively carry out its watchdog functions. Resultantly, member states that practice child labour may set a minimum working age as low as 13 years and still not adhere to it, thus perpetuating the employment of children, who in fact, are school age. Therefore, ILO needs to urgently rectify this loophole by setting a uniform minimum working age, say 18 years, if meaningful gains on banning chid labour are to be attained.

Improvements are required for industrialised and developing countries with regard to addressing some health inequalities. For example, migrant workers and refugees’ right of access to health services seem to be unfulfilled in some industrialised countries [13,14], which is a violation of the United Nations’ article 25 on the right to health for all. Still, some authors found positive associations between the poor health of immigrants and discrimination [15]. There appears to be limited literature that discusses the extent to which developing countries have complied with international conventions that abolished child labour, forced labour and bonded labour. Such a discussion is critical, particularly in the context of the current UN Sustainable Development Goal number 8 on decent work and the 2014 ILO Protocol on Forced Labour [16], which devote unequivocal emphasis to these fundamental human rights infringements.

In light of the explosive HIV/AIDs prevalence rate in some low- and middle-income countries, it is also important for the scientific community to examine how governments and employers have addressed the issues of workplace discrimination against workers with HIV/AIDs. Areas of focus may entail the adequacy and coverage of existing national legislative and policy measures. The existence of global anti-discrimination pacts [17,18] is a positive development that provides a valuable yardstick to measure the level of compliance to their provisions by member states.

To conclude, Moyo and colleagues’ findings elegantly provide a wonderful call for governments to freshen up and attend to deficits within their OHS laws. We urge responsible authorities to rectify the limitations of the national OHS laws and of the intergovernmental pacts. We also call upon industrialised nations to attend to the health needs of refugees, migrant and informal workers.
